# Localization of Brain Injuries Using Cranial Electromagnetic Fields

**DOI:** 10.7759/cureus.80518

**Published:** 2025-03-13

**Authors:** Alice S Wang, Raphia K Rahman, Paras Savla, James Brazdzionis, Tye Patchana, Imran Siddiqi, Dan E Miulli

**Affiliations:** 1 Department of Neurosurgery, Riverside University Health System Medical Center, Moreno Valley, USA; 2 Department of Neurosurgery, Arrowhead Regional Medical Center, Colton, USA

**Keywords:** atraumatic brain injury, concussion, electromagnetic field, electromagnetic field frequency, lesion localization, neural circuit, traumatic brain injury

## Abstract

Background: Atraumatic brain injury and traumatic brain injury (TBI) have been demonstrated to be associated with changes in brain electromagnetic field (EMF) activity due to alterations in the structure and function of neural circuitry. Modulation of abnormal EMF activity through EMF stimulation may promote neural regeneration and may be more beneficial when the specific change in EMF frequency that is correlated with either computed tomography (CT) imaging changes, neurological changes, or both can be precisely localized. The authors investigate the efficacy and feasibility of a noninvasive portable helmet with sensors and built-in signal generators to measure and localize specific changes with frequency and amplitude from brain EMF for both atraumatic and TBI patients.

Methods: This prospective clinical study was conducted from January 2025 to February 2025 and enrolled patients greater than 18 years old diagnosed with atraumatic and TBI, including negative image concussion. Baseline EMF activity was recorded using a helmet equipped with 20 sensor stimulators. Localization of EMF activity was determined based on sensor activity corresponding to the patient’s neurological deficits on exam and/or structural lesion(s) on CT. EMF data were collected using the DAQami software (Dataq Instruments, Akron, OH) and analyzed using fast Fourier transformation with the Igor Pro 8 software (Wavemetrics Inc., Lake Oswego, OR) to localize normal and abnormal brain EMF signals by comparing opposing and adjacent sensors.

Results: Ten patients were enrolled in this study with a mean age of 47.1 years. Mechanisms of injury included spontaneous hypertensive intracranial hemorrhage (one patient) and head trauma after motor vehicle collision (auto vs. auto; auto vs. motorcycle; and auto vs. pedestrian), dirt bike accident, and ground-level fall (nine patients). Radiographic findings included spontaneous basal ganglia hemorrhage (one patient), isolated traumatic subdural hematoma (one patient), traumatic subarachnoid hemorrhage (one patient), and no intracranial abnormalities (seven patients). Abnormal EMF activity was recorded and correlated with neurological deficits on exam, CT findings, or both, demonstrating the usefulness of EMF in localizing brain injuries.

Conclusions: These results demonstrate the efficacy and feasibility of utilizing a noninvasive portable helmet for real-time EMF recording and localization of brain abnormalities in atraumatic and TBI patients, including image-negative concussions. EMF measurements may aid in monitoring recovery after atraumatic brain injury and TBI and enable the clinician to tailor treatment plans based on the patient’s unique brain EMF patterns.

## Introduction

Atraumatic brain injury and traumatic brain injury (TBI) have been demonstrated to be associated with physical, functional, and metabolic changes in neuronal cells, neural circuitry, and neuronal pathways resulting in electromagnetic field (EMF) changes such as low-frequency amplitude increases and peak alpha wave oscillatory slowing [1-5]. The EMF of the human brain without disease of the central nervous system and following TBI can be measured in real time through the skull using a noninvasive portable helmet and EMF sensors [6-10]. To aid in recovery and rehabilitation after brain injury, using the information gained from the real-time recording of EMF, low- or high-frequency electromagnetic stimulation may be applied to increase cortical activity, specifically after localization of a brain lesion, whether structural or functional, has been determined [1-3]. In a swine model with induced TBI at a specific brain location, EMF stimulation at 2.5 and 5.5 Hz thresholds improved EMF patterns by day 7 of treatment [2]. It has previously been shown that modulation of abnormal EMF signaling may promote neural regeneration, alter the genetic expression of proteins, and reduce the detrimental effects of inflammatory cytokines and abnormal cell signaling to promote recovery in injured neuronal cells when targeted to the abnormality [4,5].

Using a cost-effective and noninvasive helmet equipped with 20 sensors, the brain EMF activity of atraumatic and TBI patients was recorded. In this study, the authors investigated the relationship among neurological deficits on exam, computed tomography (CT) findings, and EMF recordings to elucidate if EMF recordings can be used to localize the sites of brain injuries. The ability to localize brain injuries via EMF can provide an opportunity to modulate the neural circuitry via EMF stimulation precisely. The authors hypothesized that abnormal clinical and/or radiographic findings would manifest as abnormal EMF recordings with lower amplitudes in areas of brain lesions compared to higher amplitudes in areas without brain lesions.

## Materials and methods

Study design

This prospective clinical study was approved by the institution’s institutional review board (Protocol #23-58: Transcranial electromagnetic field stimulation for modulation of brain activity in patients with neurological disorders). Patients over 18 with head trauma or intracranial hematomas were included. Exclusion criteria included a Glasgow Coma Scale (GCS) of 3, contraindications for donning a helmet, such as active hemodynamic or respiratory instability, or refusal of study enrollment.

Helmet and sensor configuration

**Figure 1:**
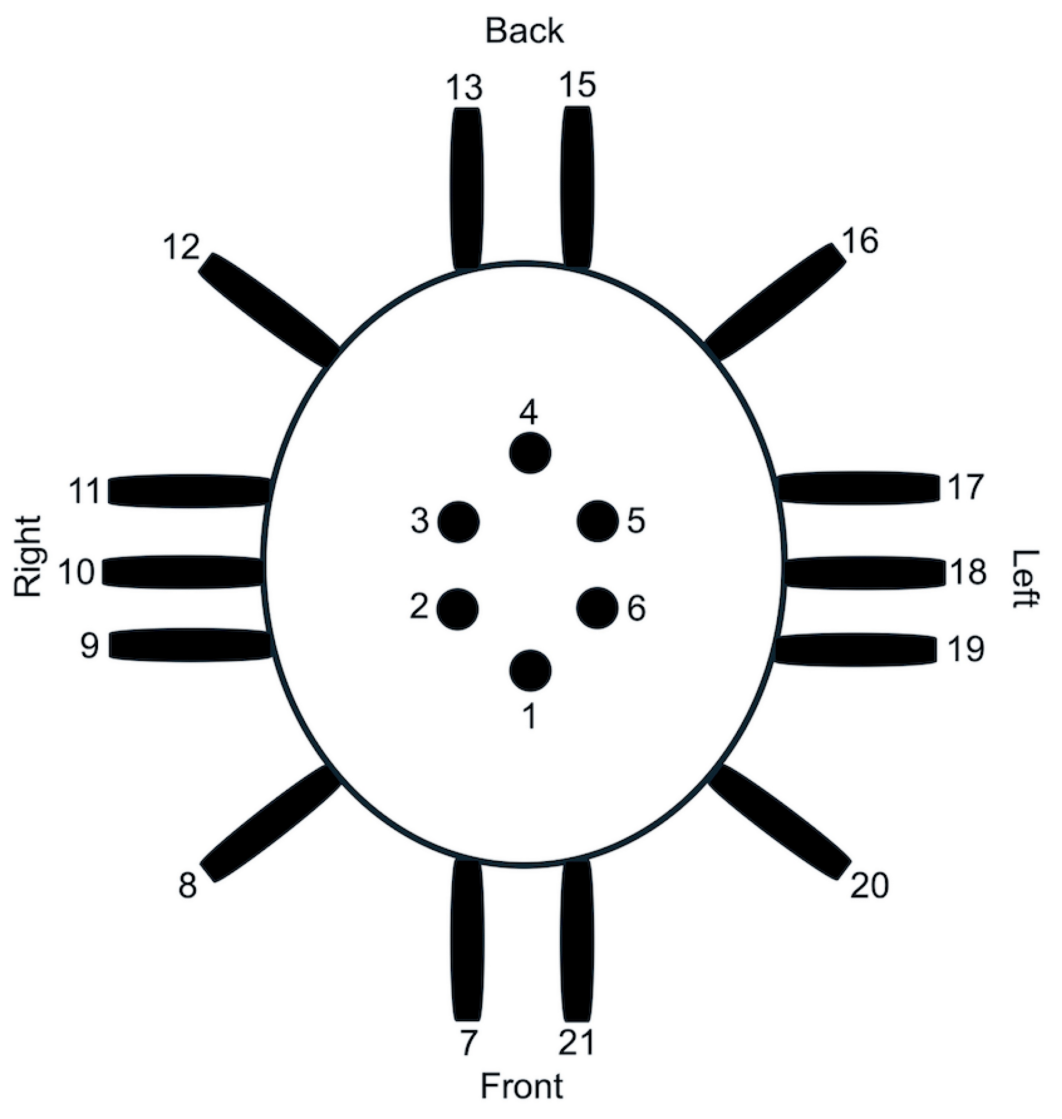
Topography of the helmet. The configuration of the 20 electromagnetic field sensors labeled from 1 to 21 (skipping 14) in the lightweight helmet is demonstrated. All sensors are wrapped with Mu-metal foil and placed 9 inches above the scalp, oriented with the positive side facing toward the scalp

Two portable racks equipped with horizontal rods and four cords were used to suspend the helmet in air, and the cords provided adjustable tension to securely hang the helmet in place just above the patient’s head. The portable helmet with shielding constrained to a dual-layered Mu-metal (MuMETAL, Magnetic Shield Corporation, Bensenville, IL) and copper layering and engineered with Mu-metal 18-inch channels to place sensors and EMF signal generators (BS-1000, Quasar Federal Systems, San Diego, CA) was built to allow for EMF recording at bedside instead of bringing the patient to a room designed for EMF recordings. The sensors and EMF signal generators were placed in a specific configuration, as shown in Figure 1. The sensor placement was as follows: sensor 1 spans the frontal lobes, sensor 2 right motor cortex and deeper structures, sensor 3 right sensory cortex and deeper structures, sensor 4 parietal lobes, sensor 5 left sensory cortex and deeper structures, sensor 6 left motor cortex and deeper structures, sensor 7 right frontal lobe, sensor 8 right frontotemporal region, sensors 9 right anterior temporal lobe, sensor 10 right temporal lobe, and sensor 11 right posterior temporal lobe, sensor 12 right parietooccipital region, sensor 13 right occipital lobe, sensor 15 left occipital lobe, sensor 16 left parietooccipital region, sensor 17 left anterior temporal lobe, sensor 18 left temporal lobe, sensor 19 left posterior temporal lobe, sensor 20 left frontotemporal region, and sensor 21 left frontal lobe (Figure 1). The sensors were placed in the channels 9 inches from the scalp, providing a 6.37° field of view. The known spatial relationship between the sensors allowed for identifying regions of overlap or opposite configurations, where sensors in opposing positions (180° from each other) were expected to demonstrate opposite polarities for a specific EMF. Each sensor was also positioned with the positive end oriented toward the scalp.

Relative angles between the sensors

EMF data from SOI and the opposing sensor (OS) are compared to assess for EMF changes. OSs are in an opposing configuration to SOI on the helmet (e.g., sensors 7 and 13 are in an opposing configuration) and typically overlap regions that are anatomically distant from the lesion site. These opposite-side sensors are likely to demonstrate the opposite polarity of the EMF from the sensor immediately over the area. Sensors in the area without overlap would not directly be influenced by the lesion unless sharing neuronal axonal circuitry; therefore, their activity is used to serve as a baseline for comparing activity from SOI overlying the lesion. Additionally, to calculate the expected change in wave amplitude based on different sensor locations, the relative angles between a pair of sensors are accounted for using the cosine (Cos) function (Table 1).

**Table 1:** The angles and cosines between pairs of sensors

Sensors	Angle in degree	Cosine (angle in degree)
1-2	20°	0.940
1-3	35°	0.819
1-4	40°	0.766
1-5	35°	0.819
1-7	44°	0.719
1-8	44°	0.719
1-9	50°	0.643
1-10	50°	0.643
1-11	60°	0.500
1-12	70°	0.342
1-13	80°	0.174
1-21	40°	0.766
2-3	20°	0.940
2-6	35°	0.819
3-4	20°	0.940
3-5	35°	0.819
4-5	20°	0.940
5-6	20°	0.940
6-1	20°	0.940
7-21	12°	0.978
8-7	30°	0.866
9-8	20°	0.940
10-9	12°	0.978
11-10	12°	0.978
12-11	20°	0.940
13-12	30°	0.866
15-13	12°	0.978
16-15	30°	0.866
17-16	20°	0.940
18-17	12°	0.978
19-18	12°	0.978
20-19	20°	0.940
21-20	30°	0.866

**Figure 2:**
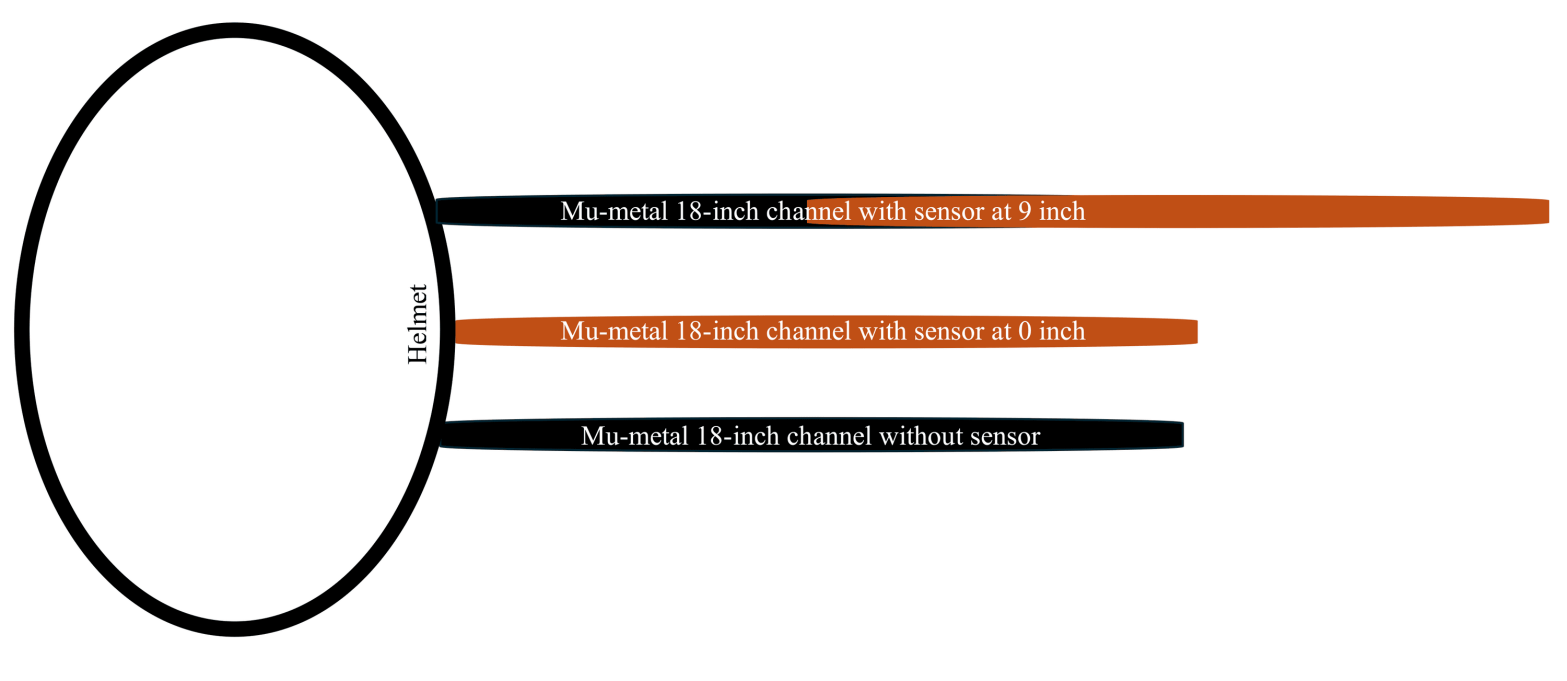
Mu-metal 18-inch channel (black) with a sensor (brown), which can be placed at 0 or 9 inches from the scalp

Based on this relationship, as the angle increases between the sensors, the wave amplitude percentage decreases. At 0° (directly above the lesion), the amplitude is at its maximum magnitude: amplitude = 100 units × cos (0°) = 100 × 1.000 = 100.0. At 45° to the lesion, the amplitude decreases: amplitude = 100 units × cos (45°) = 100 x 0.707 = 70.7. At 90° to the lesion, the amplitude drops to zero: amplitude = 100 units × cos (90°) = 100 x 0.000 = 0.0. At 180° (directly below the lesion), the amplitude flips to its negative maximum magnitude or the valley of the OS: amplitude = 100 units × cos (180°) = 100 × (-1.000) = -100.0. For example, between sensors 7 and 21 with a known angle of 12°, the amplitude would be 97.8: amplitude = 100 units × cos (12°) = 100 x 0.978 = 97.8. Finally, identifying which sensors have overlapping and nonoverlapping fields of view allows further localization. The sensors, as stated, were measured from 9 inches away from the skin, yielding a 6.37° field of view (Figure 2).

Radiographic localization of brain lesions and correlation with specific sensors

Noncontrast CT head was used to identify any structural brain lesion(s) such as subdural hematoma, intraparenchymal hemorrhage, and skull fracture. The location of the lesion was correlated with sensors that would most likely be overlying the lesion and, therefore, detect EMF activity originating from or near the lesion site. For example, a lesion in the right frontal lobe may be correlated with sensor 7 (right frontal lobe) or sensor 8 (right frontotemporal region). Sensors identified to correlate with the lesion(s) are termed sensors of interest (SOIs). Sensors opposite of these SOIs are termed OSs.

EMF data collection and analysis

Before EMF recordings from patients, the equipment, helmet, and sensors were tested with two water bottles placed inside the helmet. Peaks and valleys, not flat lines, indicated the equipment was working properly. EMF recordings were collected using the DAQami software (Dataq Instruments, Akron, OH) and analyzed using fast Fourier transformation (FFT) with the Igor Pro 8 software (Wavemetrics Inc., Lake Oswego, OR). Each EMF recording was 30 seconds. To ensure stability of data, only 20 seconds of the recording was used for analysis (data between 5 seconds after the start of recording and 5 seconds before the end of the recording). Graphs of EMF recordings were then generated using FFT with the Igor Pro 8 software. The X-axis represented frequency, and the Y-axis represented amplitude. The data recorded represent the summative amplitude within a given frequency over the 20-second period of data analysis.

After identifying the neurological pathologies and examination in multiple brain injured patients, EMF recordings were grouped into one of three categories: 0.0-4.0 Hz range was the major frequency in comatose patients (category 1), 4.0-6.0 Hz range for those with neurologic deficits of neuronal circuits (i.e., speech, motor, or sensory deficits) (category 2), and 6-12 Hz range for those suffering from concussion-like symptoms or cognitive deficits (e.g., headache, loss of consciousness, and difficulty with concentration) without basic functional neurologic deficits of category 2 on examination (category 3) [3]. These categories were formulated based on evidence that delta waves (0-4 Hz) are typically observed in states of diminished consciousness; theta wave activity (4-6 Hz) plays a role in motor function and speech processing, while alpha activity (8-13 Hz) is the hallmark electrophysiologic rhythm of the normal and awake brain [11-15]. Changes in frequencies above 12 Hz are not the subject of this paper. Each patient was assigned a category based on their neurological exam, and the EMF data within that category was analyzed and used for localization.

Within a specific category, each pair of OSs, such as sensors 7 and 13, was evaluated for peaks and valleys. Peaks were defined as the inflection point on the graph of maximal amplitude reached where the Y-value is higher than both of its immediate neighboring points, while valleys were defined as the inflection point of lowest amplitude where the Y-value is lower than both of its immediate neighboring points. Deficits in brain EMF were defined as regions where the amplitude was decreased across most sensors, which were visualized as a prominent valley or negative peak, under conditions of optimal sensor orientation. Therefore, a valley in one sensor and a peak in its OS at the same frequency indicated EMF activity localized to the sensor with the valley. Only pairs of sensors showing peaks and valleys at the same frequency were labeled SOI and OS. Other pairs of sensors were labeled as OSs. There may be multiple pairs of SOI and OS; the pair selected for focus, termed targeted SOI and OS, correlated with the patient’s clinical presentation and/or radiographic findings.

## Results

A total of 10 patients with atraumatic brain injury and TBI, including image-negative concussion, were enrolled between January 2025 and February 2025. The patients’ demographics, clinical characteristics, radiographic findings, and EMF recordings were collected (Table 2). The mean age was 47.1 years. Mechanisms of injury included spontaneous hypertensive intracranial hemorrhage (one patient) and head trauma after motor vehicle collision (auto vs. auto; auto vs. motorcycle; and auto vs. pedestrian), dirt bike accident, and ground-level fall (nine patients). Radiographic findings included spontaneous basal ganglia hemorrhage (one patient), isolated traumatic subdural hematoma (one patient), traumatic subarachnoid hemorrhage (one patient), and no CT image intracranial abnormalities (seven patients). Abnormal EMF activity was recorded and observed to be correlated with either the neurological deficits on exam, CT findings, or both, demonstrating successful localization of brain injuries via EMF.

**Table 2:** Patient demographics, clinical presentations, computed tomography findings, and electromagnetic field findings ^*^The hypothesized sensor of interest and OS found on electromagnetic field findings. LOC: loss of consciousness; GCS: Glasgow Coma Scale; SOI: sensor of interest; OS: opposing sensor

Patient	Age (years)/sex	Mechanism of injury	Chief complaint	Clinical findings	Computed tomography findings	Category (frequency range in Hertz)	Hypothesized SOI (OS)	Electromagnetic field findings SOI (OS)
1	38/male	Motor vehicle collision	LOC	GCS15, left frontal scalp hematoma	No intracranial abnormalities	3 (6-12)	21 (15) and 20 (12)^*^	2 (5), 8 (16), and 20 (12)^*^
2	23/male	Motorcycle accident	Right posterior temporal headache, helmeted, LOC	GCS15, headache 2/10	No intracranial abnormalities	3 (6-12)	11 (17)^*^	9 (19), 10 (18), 11 (17)^*^, and 12 (20)
3	23/female	Motor vehicle collision	Right frontotemporal headache, no LOC	GCS15, headache 5/10	No intracranial abnormalities	3 (6-12)	7 (13) and 8 (16)^*^	4 (1), 7 (13), 8 (16)^*^, and 10 (18)
4	36/male	Dirt bike accident	Right shoulder pain, headstrike, no LOC	GCS15	No intracranial abnormalities	3 (6-12)	12 (20)^* ^and 13 (7)	6 (3), 11 (17), and 12 (20)^*^
5	81/female	Ground level fall	Bilateral frontal headache, headstrike, LOC	GCS15, bilateral forehead ecchymosis	Right subarachnoid hemorrhage in the ambient cistern	3 (6-12)	7 (13) and 21 (15)^*^	5 (2), 7 (13), 11 (17), and 21 (15)^*^
6	46/male	Motor vehicle collision	Left top headache, headstrike, LOC	GCS15, headache 8/10	No intracranial abnormalities	3 (6-12)	6 (3)^*^	6 (3)^*^, 13 (7), 16 (8), 9 (19), 12 (20), and 15 (21)
7	81/male	Ground level fall	Left temporal headache, headstrike, LOC	GCS15, headache 10/10, right frontal scalp hematoma	No intracranial abnormalities	3 (6-12)	17 (11)^*^, 18 (10), 19 (9), and 7 (13)	1 (4), 17 (11)^*^, and 18 (10)
8	57/male	Motor vehicle collision	Right posterior temporal headache, headstrike, no LOC	GCS15, headache 6/10	Anterior falx subdural hematoma	3 (6-12)	11 (17)^*^	11 (17)^*^
9	62/male	Hypertension	Hard to speak, right arm weakness, no LOC	GCS15, expressive aphasia, 4/5 right upper extremity motor strength, right pronator drift	Acute left basal ganglia hemorrhage	2 (4-6)	6 (3) and 5 (2)^*^	4 (1), 5 (2)^*^, and 12 (20)
10	24/male	Motorcycle accident	Left frontal headache, headstrike, LOC	GCS15, headache 7/10	No intracranial abnormalities	3 (6-12)	21 (15)^*^	21 (15)^*^

Patient 1

**Figure 3:**
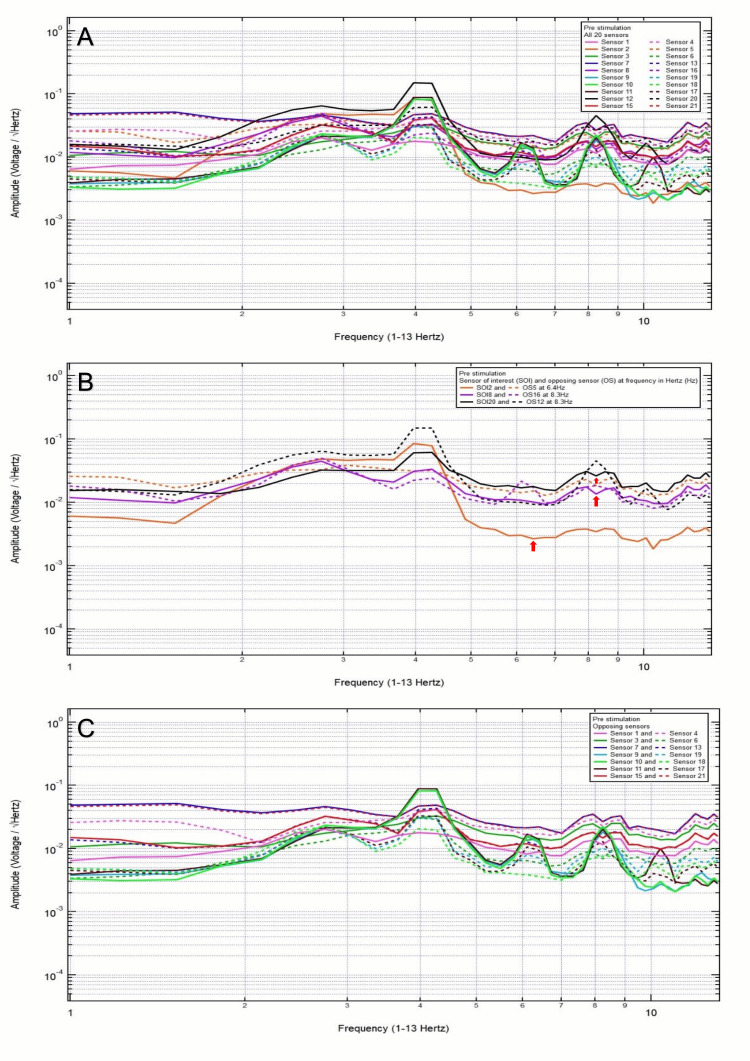
Patient 1 EMF EMF showed peaks and valleys between 6 and 12 Hz (category 3) (A). Within category 3, each pair of the following sensors showed a valley and a peak, respectively, at the same frequency: SOI2 (right motor cortex and deeper structures) and OS5 (left sensory cortex and deeper structures) at 6.4 Hz, SOI8 (right frontotemporal region) and OS16 (left parietooccipital region) at 8.3 Hz, and SOI20 (left frontotemporal region) and OS12 (right parietooccipital region) at 8.3 Hz with red arrows pointing at all the above frequencies (B). Each pair of sensors that did not show a peak and a valley at the same frequency was termed only OSs, not the SOI (C) EMF: electromagnetic field; SOI: sensors of interest; OS: opposing sensor

A 38-year-old male patient presented with no chief complaint of headache and dizziness after a motor vehicle collision with loss of consciousness. Clinically, the patient was GCS15 with a small left frontal scalp hematoma. CT head was negative for intracranial abnormalities. Based on the clinical and radiographic findings, the patient was assigned to category 3, and the hypothesized SOIs were 21 and 20, which spanned the left frontal lobe and the left frontotemporal region, respectively. His baseline EMF showed peaks and valleys between 6 and 12 Hz (category 3) (Figure 3A). Within category 3, each pair of the following sensors showed a valley and a peak, respectively, at the same frequency: SOI2 (right motor cortex and deeper structures) and OS5 (left sensory cortex and deeper structures) at 6.4 Hz; SOI8 (right frontotemporal region) and OS16 (left parietooccipital region) at 8.3 Hz; and SOI20 (left frontotemporal region) and OS12 (right parietooccipital region) at 8.3 Hz (Figure 3B). Each pair of sensors that did not show a peak and a valley at the same frequency was termed only OSs, not the SOIs (Figure 3C).

Patient 2

**Figure 4:**
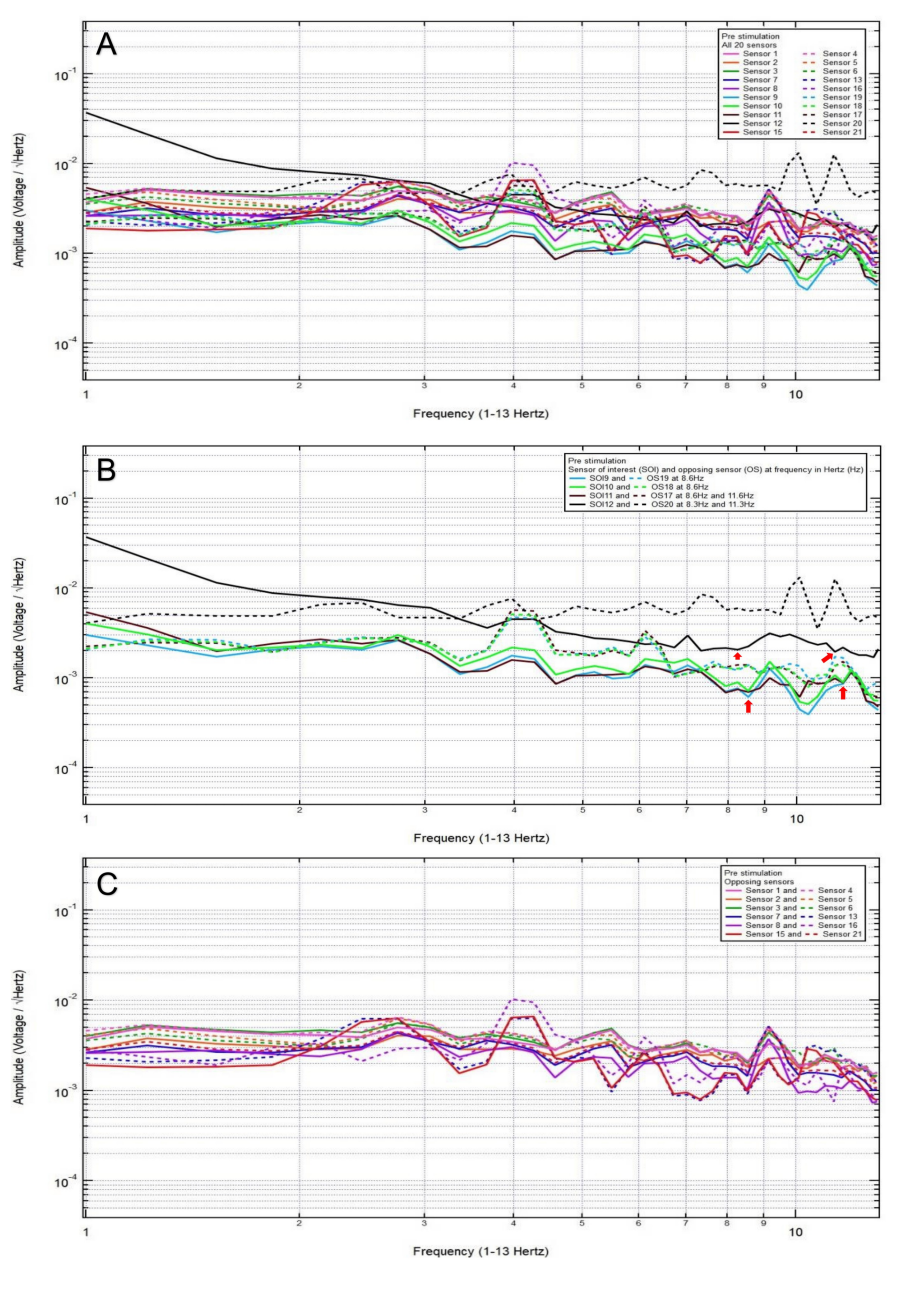
Patient 2 EMF EMF showed peaks and valleys between 6 and 12 Hz (category 3) (A). Within category 3, each pair of the following sensors showed a valley and a peak, respectively, at the same frequency: SOI9 (right anterior temporal region) and OS19 (left anterior temporal region) at 8.6 Hz, SOI10 (right temporal region) and OS18 (left temporal region) at 8.6 Hz, SOI11 (right posterior temporal region) and OS17 (left posterior temporal region) at 8.6 and 11.6 Hz, and SOI12 (right parietooccipital region) and OS20 (left frontotemporal region) at 8.3 and 11.3 Hz with red arrows pointing at all the above frequencies (B). Each pair of sensors that did not show a peak and a valley at the same frequency was termed only OS, not the SOI (C) EMF: electromagnetic field; SOI: sensor of interest; OS: opposing sensor

A 23-year-old male patient presented with a chief complaint of right posterior temporal headache after a motorcycle accident, helmeted, with loss of consciousness. Clinically, the patient was GCS15 and had a right posterior temporal headache, 2/10 pain. CT head was negative for intracranial abnormalities. Based on clinical and radiographic findings, the patient was assigned to category 3, and the hypothesized SOI was 11, which spanned the right posterior temporal region. His baseline EMF showed peaks and valleys between 6 and 12 Hz (category 3) (Figure 4A). Within category 3, each pair of the following sensors showed a valley and a peak, respectively, at the same frequency: SOI9 (right anterior temporal region) and OS19 (left anterior temporal region) at 8.6 Hz, SOI10 (right temporal region) and OS18 (left temporal region) at 8.6 Hz, SOI11 (right posterior temporal region) and OS17 (left posterior temporal region) at 8.6 and 11.6 Hz, and SOI12 (right parietooccipital region) and OS20 (left frontotemporal region) at 8.3 and 11.3 Hz (Figure 4B). Each pair of sensors that did not show a peak and a valley at the same frequency was termed only OS, not SOI (Figure 4C).

Patient 3

**Figure 5:**
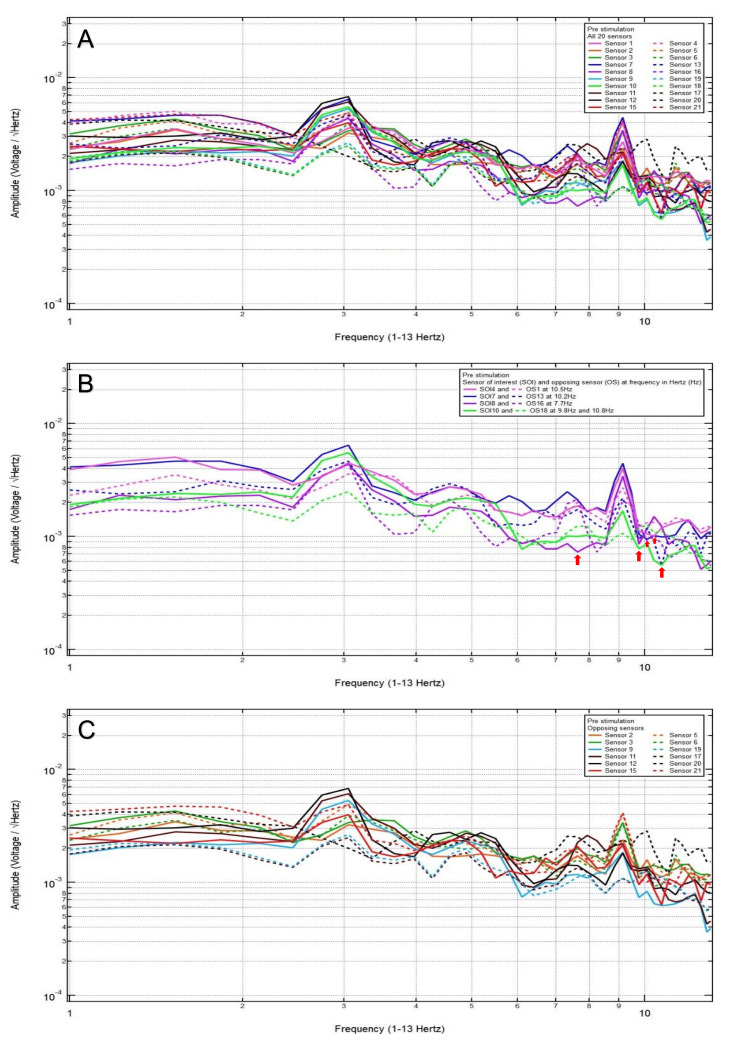
Patient 3 EMF EMF showed peaks and valleys between 6 and 12 Hz (category 3) (A). Within category 3, each pair of the following sensors showed a valley and a peak at the same frequency: SOI4 (parietal lobes) and OS1 (frontal lobes) at 10.5 Hz, SOI7 (right frontal lobe) and OS13 (right occipital lobe) at 10.2 Hz, SOI8 (right frontotemporal region) and OS16 (left parietooccipital region) at 7.7 Hz, and SOI10 (right temporal region) and OS18 (left temporal region) at 9.8 and 10.8 Hz with red arrows pointing at all the above frequencies (B). Each pair of sensors that did not show a peak and a valley at the same frequency was termed only OS without being SOI (C) EMF: electromagnetic field; SOI: sensor of interest; OS: opposing sensor

A 23-year-old left-handed female patient presented with a chief complaint of right frontotemporal headache after hitting the right side of her head in a motor vehicle collision without loss of consciousness. Clinically, the patient was GCS15 and had right frontotemporal headache, 5/10 pain. CT head was negative for intracranial abnormalities. Based on clinical and radiographic findings, the patient was assigned to category 3, and the hypothesized SOI were 7 and 8, which spanned the right frontal lobe and the right frontotemporal region. Her baseline EMF showed peaks and valleys between 6 and 12 Hz (category 3) (Figure 5A). Within category 3, each pair of the following sensors showed a valley and a peak at the same frequency: SOI4 (parietal lobes) and OS1 (frontal lobes) at 10.5 Hz; SOI7 (right frontal lobe) and OS13 (right occipital lobe) at 10.2 Hz; SOI8 (right frontotemporal region) and OS16 (left parietooccipital region) at 7.7 Hz; and SOI10 (right temporal region) and OS18 (left temporal region) at 9.8 and 10.8 Hz (Figure 5B). Each pair of sensors that did not show a peak and a valley at the same frequency was termed only OS without being SOI (Figure 5C).

Patient 4

**Figure 6:**
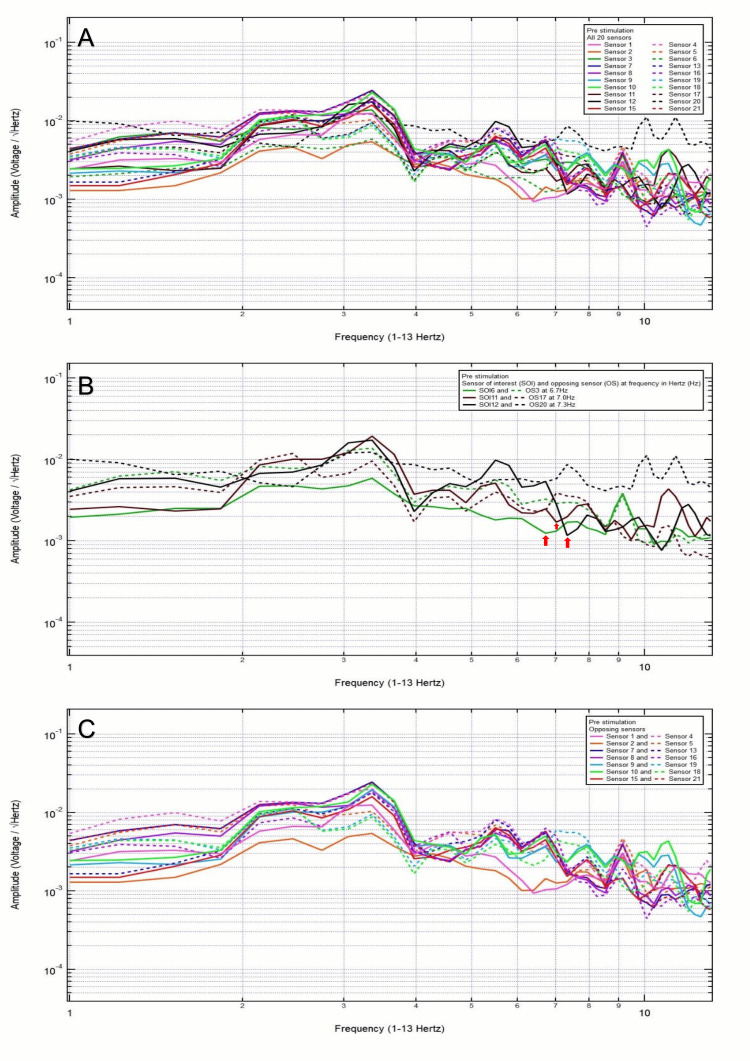
Patient 4 EMF EMF showed peaks and valleys between 6 and 12 Hz (category 3) (A). Within category 3, each pair of the following sensors showed a valley and a peak at the same frequency: SOI6 (left motor cortex and deeper structures) and OS3 (right sensory cortex and deeper structures) at 6.7 Hz, SOI11 (right posterior temporal region) and OS17 (left posterior temporal region) at 7.0 Hz, and SOI12 (right parietooccipital region) and OS20 (left frontotemporal region) at 7.3 Hz with red arrows pointing at all the above frequencies (B). Each pair of sensors that did not show a peak and a valley at one frequency was termed OS without being the SOI (C) EMF: electromagnetic field; SOI: sensor of interest; OS: opposing sensor

A 36-year-old male patient presented with a chief complaint of right shoulder pain after a dirt bike accident, helmeted, with headstrike, and without loss of consciousness. Clinically, the patient was GCS15. CT head was negative for intracranial abnormalities. Based on clinical and radiographic findings, the patient was assigned to category 3, and the hypothesized SOIs were 12 and 13, which spanned the right parietooccipital region and the right occipital region, respectively, given that these regions were closest to his right shoulder pain. His baseline EMF showed peaks and valleys between 6 and 12 Hz (category 3) (Figure 6A). Within category 3, each pair of the following sensors showed a valley and a peak at the same frequency: SOI6 (left motor cortex and deeper structures) and OS3 (right sensory cortex and deeper structures) at 6.7 Hz, SOI11 (right posterior temporal region) and OS17 (left posterior temporal region) at 7 Hz, and SOI12 (right parietooccipital region) and OS20 (left frontotemporal region) at 7.3 Hz (Figure 6B). Each pair of sensors that did not show a peak and a valley at one frequency was termed OS without being the SOI (Figure 6C).

Patient 5

**Figure 7:**
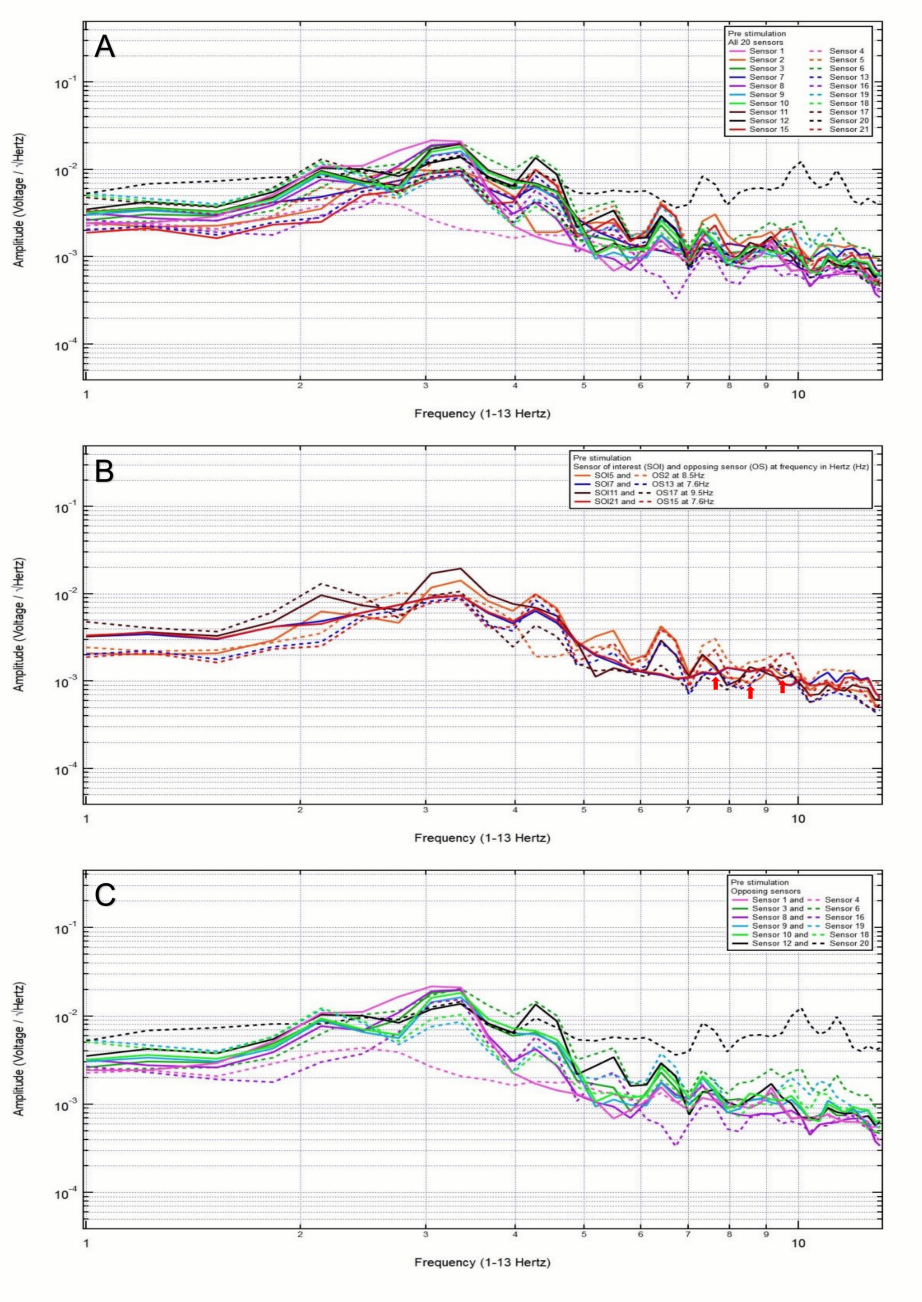
Patient 5 EMF EMF showed peaks and valleys between 6 and 12 Hz (category 3) (A). Within category 3, each pair of the following sensors showed a valley and a peak at the same frequency: SOI5 (left sensory cortex and deeper structures) and OS2 (right motor cortex and deeper structures) at 8.5 Hz, SOI7 (right frontal lobe) and OS13 (right occipital lobe) at 7.6 Hz, SOI11 (right posterior temporal lobe) and OS17 (left posterior temporal lobe) at 9.5 Hz, and SOI21 (left frontal lobe) and OS15 (left occipital lobe) at 7.6 Hz with red arrows pointing at all the above frequencies (B). Each pair of sensors that did not show a peak and a valley at one frequency was termed OS without being SOI (C) EMF: electromagnetic field; SOI: sensor of interest; OS: opposing sensor

An 81-year-old female patient presented with a chief complaint of bilateral frontal headache, 1/10 in pain, after a ground-level fall, with headstrike and loss of consciousness. Clinically, the patient was GCS15 with bilateral forehead ecchymosis. CT head showed right subarachnoid hemorrhage in the ambient cistern. Based on the clinical and radiographic findings, the patient was assigned to category 3, and the hypothesized SOIs were 7 and 21, which spanned the right frontal lobe and left frontal lobe, respectively. Her baseline EMF showed peaks and valleys between 6 and 12 Hz (category 3) (Figure 7A). Within category 3, each pair of the following sensors showed a valley and a peak at the same frequency: SOI5 (left sensory cortex and deeper structures) and OS2 (right motor cortex and deeper structures) at 8.5 Hz, SOI7 (right frontal lobe) and OS13 (right occipital lobe) at 7.6 Hz, SOI11 (right posterior temporal lobe) and OS17 (left posterior temporal lobe) at 9.5 Hz, and SOI21 (left frontal lobe) and OS15 (left occipital lobe) at 7.6 Hz (Figure 7B). Each pair of sensors that did not show a peak and a valley at one frequency was termed OS without being SOI (Figure 7C).

Patient 6

**Figure 8:**
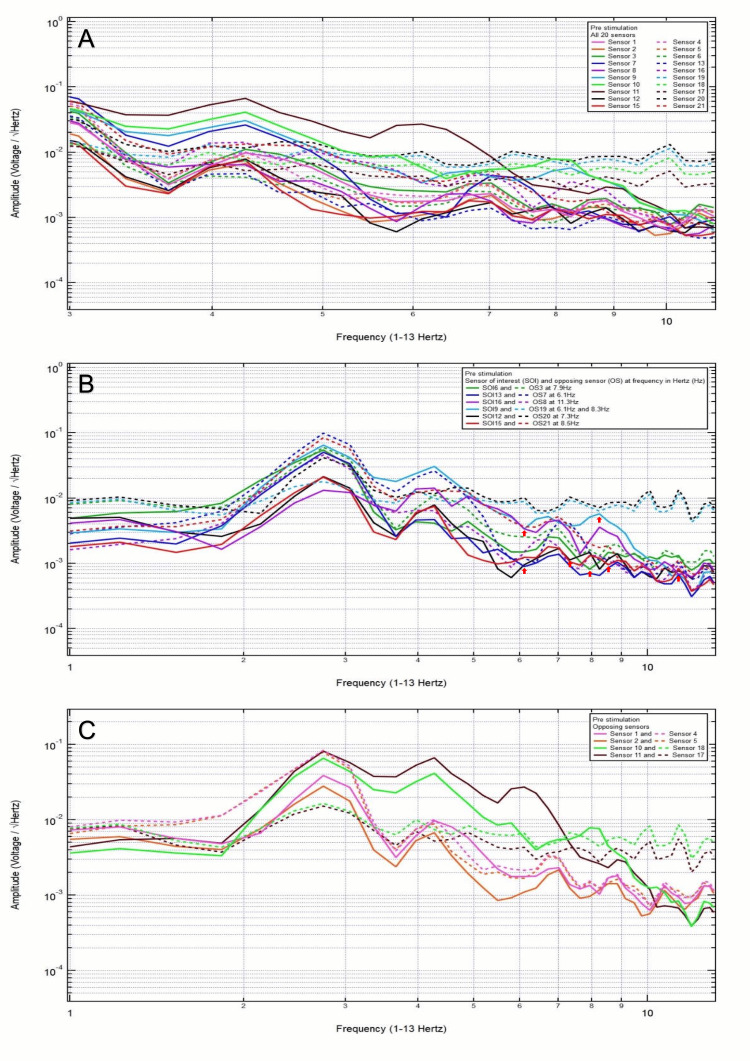
Patient 6 EMF EMF showed peaks and valleys between 6 and 12 Hz (category 3) (A). Within category 3, each pair of the following sensors showed a valley and a peak at the same frequency: SOI6 (left motor cortex and deeper structures) and OS3 (right sensory cortex and deeper structures) at 7.9 Hz, SOI13 (right occipital lobe) and OS7 (right frontal lobe) at 6.1 Hz, SOI16 (left parietooccipital lobe and OS8 (right frontotemporal lobe) at 11.3 Hz, SOI9 (right anterior temporal lobe) and OS19 (left anterior temporal lobe) at 6.1 and 8.3 Hz, SOI12 (right parietooccipital region) and OS20 (left frontotemporal region) at 7.3 Hz, and SOI15 (left occipital lobe) and OS21 (left frontal lobe) at 8.5 Hz with red arrows pointing at all the above frequencies (B). Each pair of sensors that did not show a peak and a valley at one frequency was termed only OS without being SOI (C) EMF: electromagnetic field; SOI: sensor of interest; OS: opposing sensor

A 46-year-old left-handed male patient presented with a chief complaint of left top headache, 8/10 pain, after a motor vehicle collision, with headstrike and loss of consciousness. Clinically, the patient was GCS15 and had left eye ecchymosis and facial swelling. CT head showed no intracranial abnormalities. Based on clinical and radiographic findings, the patient was assigned to category 3, and the hypothesized SOIs were 6, 21, and 20, which spanned the left motor cortex/deeper structures, the left frontal lobe, and the left frontotemporal lobe, respectively. His baseline EMF showed peaks and valleys between 6 and 12 Hz (category 3) (Figure 8A). Within category 3, each pair of the following sensors showed a valley and a peak at the same frequency: SOI6 (left motor cortex and deeper structures) and OS3 (right sensory cortex and deeper structures) at 7.9 Hz, SOI13 (right occipital lobe) and OS7 (right frontal lobe) at 6.1 Hz, SOI16 (left parietooccipital lobe and OS8 (right frontotemporal lobe) at 11.3 Hz, SOI9 (right anterior temporal lobe) and OS19 (left anterior temporal lobe) at 6.1 Hz and 8.3 Hz, SOI12 (right parietooccipital region) and OS20 (left frontotemporal region) at 7.3 Hz, and SOI15 (left occipital lobe) and OS21 (left frontal lobe) at 8.5 Hz (Figure 8B). Each pair of sensors that did not show a peak and a valley at one frequency was termed only OS without being SOI (Figure 8C).

Patient 7

**Figure 9:**
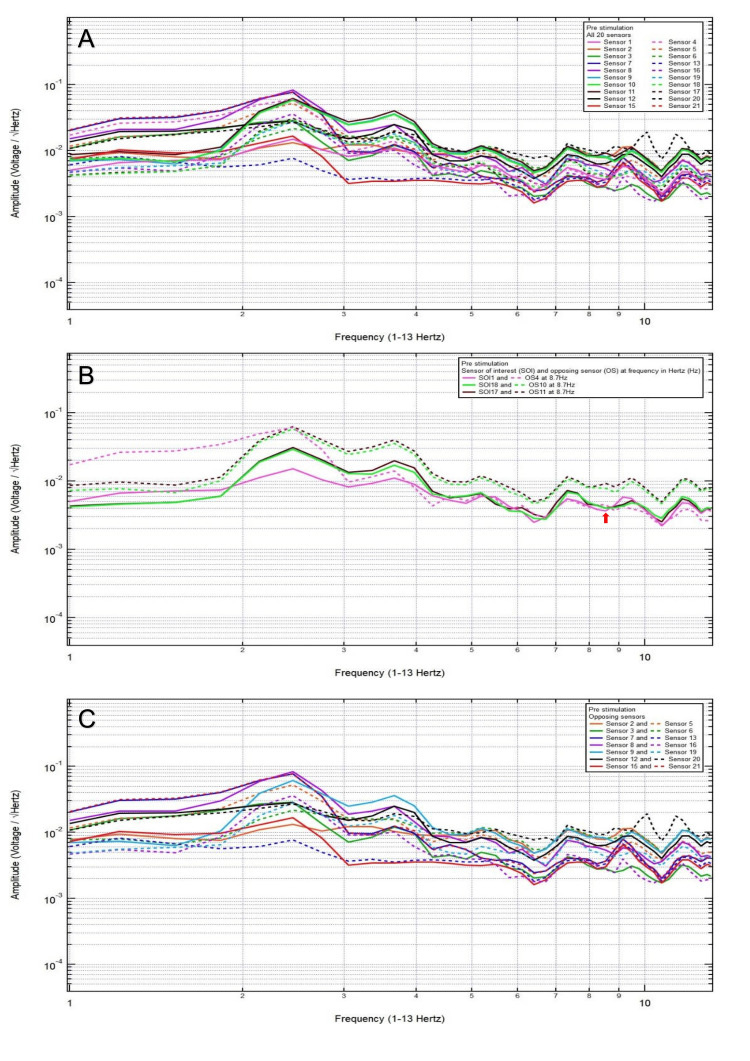
Patient 7 EMF EMF showed peaks and valleys between 6 and 12 Hz (category 3) (A). Within category 3, each pair of the following sensors showed a valley and a peak at the same frequency: SOI1 (frontal lobes) and OS4 (parietal lobes) at 8.7 Hz, SOI17 (left posterior temporal region) and OS11 (right posterior temporal region) at 8.7 Hz, and SOI18 (left temporal region) and OS10 (right temporal region) at 8.7 Hz. with the red arrow pointing at the frequency (B). Each pair of sensors that did not show a peak and a valley at one frequency was termed OS without SOI (C) EMF: electromagnetic field; SOI: sensor of interest; OS: opposing sensor

An 81-year-old male patient presented with a chief complaint of left temporal headache, 10/10 pain, after a ground-level fall, with headstrike and loss of consciousness. Clinically, the patient was GCS15 and had a large right frontal scalp hematoma. CT head was negative for intracranial abnormalities. Based on the clinical and radiographic findings, the patient was assigned to category 3, and the hypothesized SOI included 17, 18, 19, and 7, which spanned the left posterior temporal region, left temporal region, left anterior temporal region, and right frontal region, respectively. His baseline EMF showed peaks and valleys between 6 and 12 Hz (category 3) (Figure 9A). Within category 3, each pair of the following sensors showed a valley and a peak at the same frequency: SOI1 (frontal lobes) and OS4 (parietal lobes) at 8.7 Hz, SOI17 (left posterior temporal region) and OS11 (right posterior temporal region) at 8.7 Hz, and SOI18 (left temporal region) and OS10 (right temporal region) at 8.7 Hz. (Figure 9B). Each pair of sensors that did not show a peak and a valley at one frequency was termed OS without SOI (Figure 9C).

Patient 8

**Figure 10:**
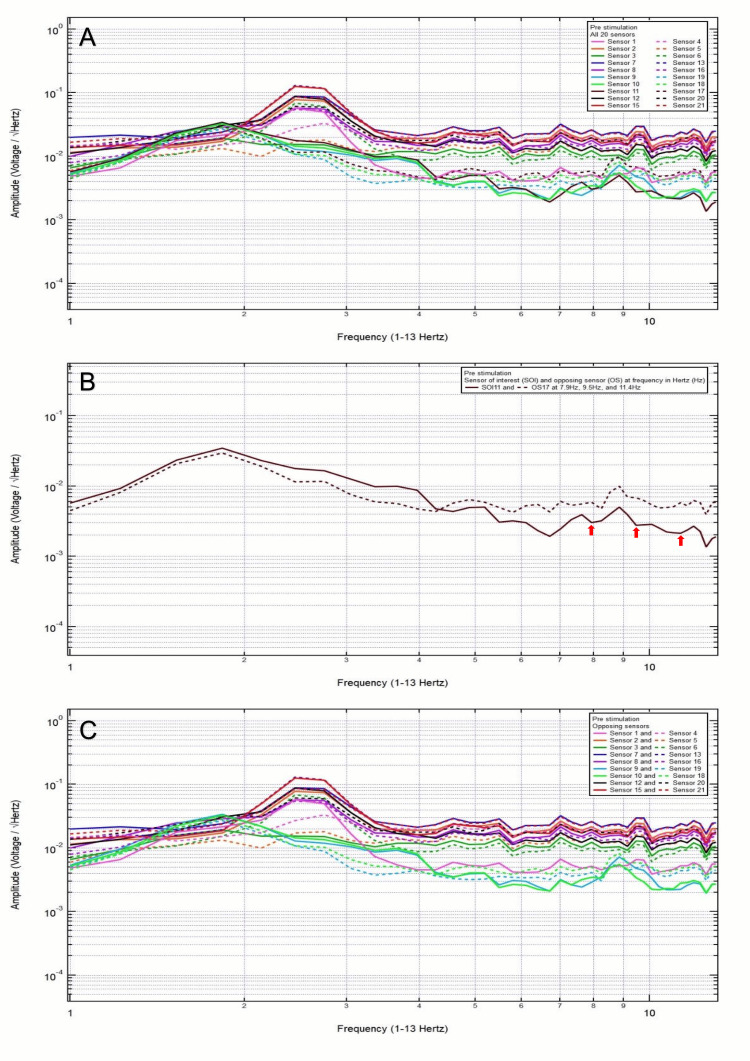
Patient 8 EMF EMF showed peaks and valleys between 6 and 12 Hz (category 3) (A). Within category 3, the following pair of sensors showed a valley and a peak at the same frequency: SOI11 (right posterior temporal region) and OS17 (left posterior temporal region) at 7.9 and 9.5 Hz, and 11.4 Hz with red arrows pointing at all the above frequencies (B). Each pair of sensors that did not show a peak and a valley at one frequency was termed as OS without being SOI (C) EMF: electromagnetic field; SOI: sensor of interest; OS: opposing sensor

A 57-year-old right-handed male patient presented with a chief complaint of right posterior temporal headache after he fell back with a headstrike after being hit by a truck from the left side without loss of consciousness. Clinically, the patient was GCS15 and had right posterior temporal headache, 6/10 pain. CT head showed anterior falx subdural hematoma without midline shift. Based on clinical and radiographic findings, the patient was assigned to category 3, and the hypothesized SOI was 11, which spanned the right posterior temporal region. His baseline EMF showed peaks and valleys between 6 and 12 Hz (category 3) (Figure 10A). Within category 3, the following pair of sensors showed a valley and a peak at the same frequency: SOI11 (right posterior temporal region) and OS17 (left posterior temporal region) at 7.9, 9.5, and 11.4 Hz (Figure 10B). Each pair of sensors that did not show a peak and a valley at one frequency was termed OS without being SOI (Figure 10C).

Patient 9

**Figure 11:**
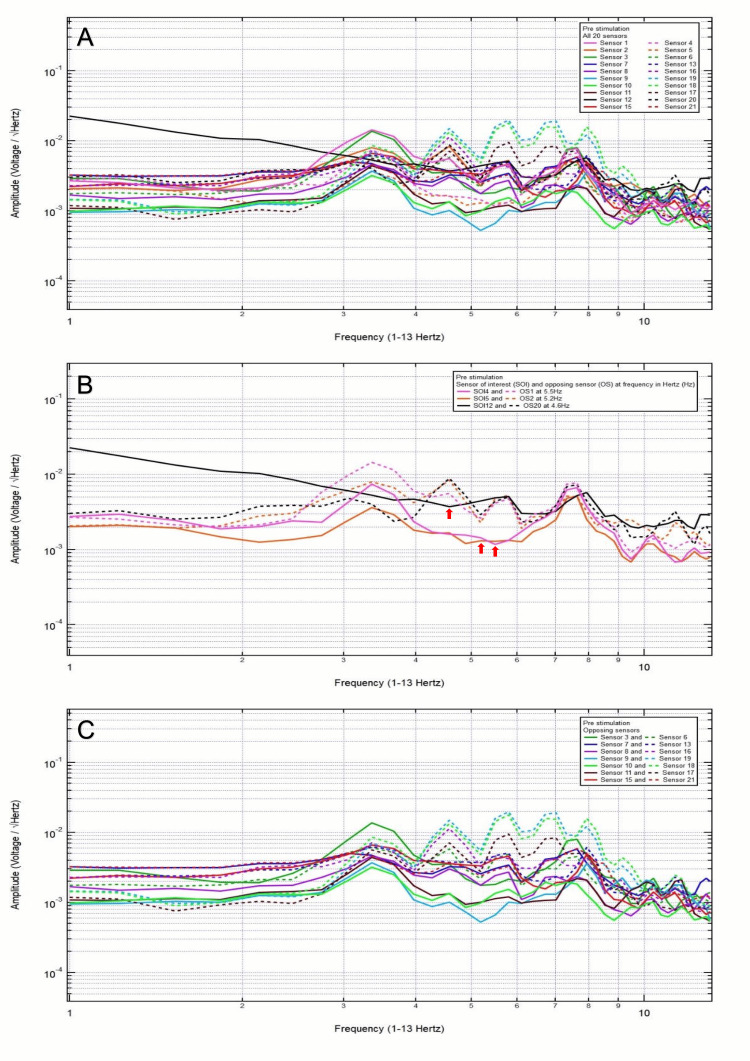
Patient 9 EMF EMF showed peaks and valleys between 4 and 6 Hz (category 2) (A). Within category 2, each pair of the following sensors showed a valley and a peak, respectively, at the same frequency: SOI4 (parietal lobes) and OS1 (frontal lobes) at 5.5 Hz, SOI5 (left sensory cortex and deeper structures) and OS2 (right motor cortex and deeper structures) at 5.2 Hz, and SOI12 (right parietal lobe) and OS20 (left frontal lobe) at 4.6 Hz with red arrows pointing at all the above frequencies (B). Each pair of sensors that did not show a peak and a valley at one frequency was termed as OS without being SOI (C) EMF: electromagnetic field; SOI: sensor of interest; OS: opposing sensor

A 62-year-old male patient presented with a chief complaint of acute aphasia and right upper arm weakness. Clinically, the patient was GCS15 and had expressive aphasia, 4/5 on the right upper extremity, and right pronator drift. CT head showed acute left basal ganglia hemorrhage. Based on the clinical and radiographic findings, the patient was assigned to category 2, and the hypothesized SOIs were 5 and 6, which spanned the left sensory cortex/deeper structures and left motor cortex/deeper structures, respectively. His baseline EMF showed peaks and valleys between 4 and 6 Hz (category 2) (Figure 11A). Within category 2, each pair of the following sensors showed a valley and a peak, respectively, at the same frequency: SOI4 (parietal lobes) and OS1 (frontal lobes) at 5.5 Hz, SOI5 (left sensory cortex and deeper structures) and OS2 (right motor cortex and deeper structures) at 5.2 Hz, and SOI12 (right parietal lobe) and OS20 (left frontal lobe) at 4.6 Hz (Figure 11B). Each pair of sensors that did not show a peak and a valley at one frequency was termed OS without being SOI (Figure 11C).

Patient 10

**Figure 12:**
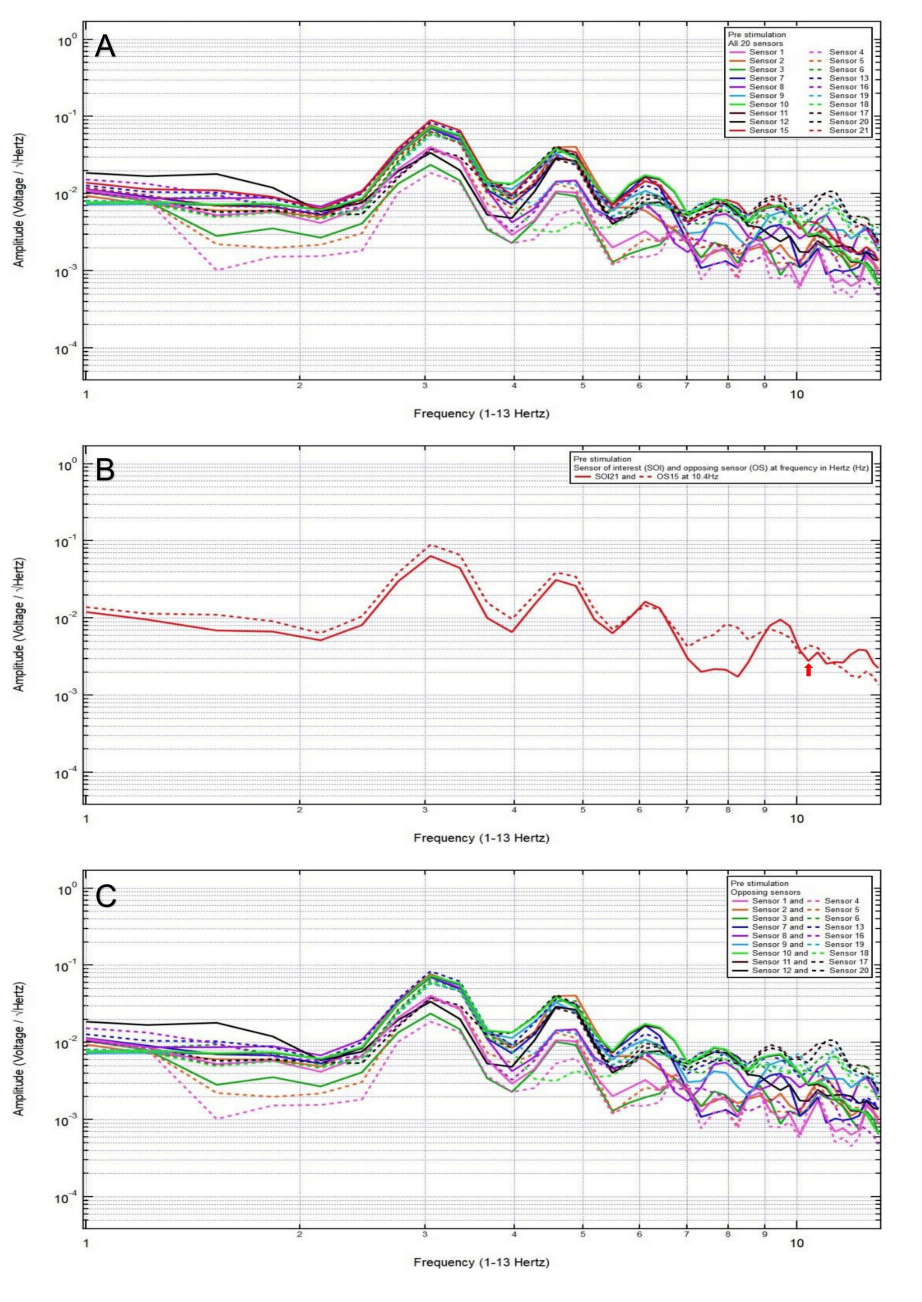
Patient 10 EMF EMF showed peaks and valleys between 4 and 6 Hz (category 2) (A). Within category 2, the following pair of sensors showed a valley and a peak, respectively, at the same frequency: SOI8 (right frontotemporal region) and OS16 (left parietooccipital region) at 5.8Hz with the red arrow pointing at the frequency (B). Each pair of sensors that did not show a peak and a valley at one frequency was termed OSs (C) EMF: electromagnetic field; SOI: sensor of interest; OS: opposing sensor

A 24-year-old male patient presented with a chief complaint of left frontal headache, 7/10 pain, after a motorcycle accident, helmeted and with loss of consciousness. Clinically, the patient was GCS15. CT head showed no intracranial abnormalities. Based on the clinical and radiographic findings, the patient was assigned to category 3, and the hypothesized SOI was 8, which spanned the right frontal lobe. His baseline EMF showed peaks and valleys between 6 and 12 Hz (category 3) (Figure 12A). Within category 3, the following pair of sensors showed a valley and a peak, respectively, at the same frequency: SOI8 (right frontotemporal region) and OS16 (left parietooccipital region) at 10.4Hz (Figure 12B). Each pair of sensors that did not show a peak and a valley at one frequency was termed OSs (Figure 12C).

## Discussion

Atraumatic brain injury and TBI patients showed brain EMF changes [1-5]. Negative valleys (or low amplitude) in EMF recordings suggest potential deficits in brain EMF activity. These negative valleys, typically observed within specific frequency ranges, were usually found in sensors corresponding to suspected lesion sites identified based on clinical and/or radiographic findings. There were additional OSs in other categories without imaging and clinical deficits. These OSs demonstrating negative valleys may correspond to clinical changes not picked up using the current methodology. For example, clinical changes may be appreciated in standardized testing of cognition, sleep, and behavior, which was not performed in this study. This localization process required data analysis from sensors spanning the suspected lesion sites, opposite sensors, adjacent sensors, sensors within and outside the field of view, and the change in the field of view based on focusing the sensor toward or away from the patient. The use of multiple sensors and their geometry was crucial to discern whether any observed abnormalities in EMF waveforms were related to the identified lesion or instead due to noise, global EMF activity, or unrelated fluctuations as evidenced in adjacent sensors, opposite sensors, and sensors not in the area of the lesion. This provided an objective baseline for comparison and helped to locate brain lesions by demonstrating distinct differences in wave amplitude patterns between lesion-adjacent sensors and their OSs. OSs helped to confirm that the observed deficits in EMF activity were directly related to the presence of structural or functional lesions and also to enhance the reliability of this localization technique.

Using clinical presentations and radiographic findings, the SOI was hypothesized. SOI overlying lesions displayed opposite polarity wave amplitudes in comparison to their OSs. For example, in patient 1 with left frontal scalp hematoma and no CT imaged intracranial abnormalities, a prominent valley or deficit in brain EMF was observed in SOI20, which spanned the left frontotemporal region, and a prominent peak was observed in its OS12 at 8.3 Hz. In another example, in patient 9 with spontaneous hypertensive basal ganglia hemorrhage, SOI5, which captured deeper structures, displayed a lower amplitude than its OS2 at 5.2 Hz.

The opposite sensor will not only record the opposite polarity of the EMF amplitude, if present, but it will also record all net positive amplitudes in the region of interest. Therefore, the OS recording may not exactly mirror the negative valley in amplitude or overall shape. This differential pattern of EMF activity, with lesion-adjacent sensors displaying lower amplitude values, strongly suggests that these regions were directly impacted by injury. The sensors that did not overlap with the lesion had normal or higher amplitude values, confirming that the observed effects in EMF were specific to the lesion site. The EMF data supported the hypothesized SOI.

Moreover, EMF recordings showed additional SOI in addition to the hypothesized ones. First, sensors near the SOI shared some level of overlap; therefore, they were able to detect abnormal EMF activities. Second, as previously discussed, additional SOI at other locations may be secondary to coup-contrecoup injury. Third, patients may not recall the specific locations of head trauma and may be asymptomatic by the methods used. Therefore, the patient may not complain of, for example, headache, in that specific location abnormalities, or of changes with sleep, behavior, or anxiety. Then, the sensor spanning this specific location would not have been considered a hypothesized SOI. Furthermore, if the CT head showed no intracranial abnormalities, then there would not be a hypothesized SOI corresponding to the structural lesion. Therefore, it was unsurprising that the EMF recordings showed more SOI than the hypothesized ones. EMF recording provides an insight into changes in the neuronal cells, neural circuitry, and neuronal pathways underlying the subclinical symptoms and subradiographic changes that would have been missed based solely on clinical and radiographic findings.

The data showed that this localization technique, using a portable lightweight helmet in the inpatient or outpatient setting, can detect structural and functional brain lesions in real time, especially in cases where conventional imaging may be inconclusive or insufficient. Although traditional imaging techniques such as CT provide high-resolution structural images, functional alterations in neural circuitry are not captured. Real-time measurement of neural EMF enables observation of electromagnetic changes in injured brain regions, which cannot be detected using CT imaging methods, particularly in cases of concussion where structural abnormalities are not always visible. The diagnosis of concussion (often characterized as a mild TBI with symptoms including confusion, amnesia, and or loss of consciousness) may be challenging because findings from clinical imaging in sports-related concussion are typically negative [16,17]. Delayed imaging findings can include cytotoxic and vasogenic edema, and pathologic changes may be detected on magnetic resonance angiography diffusion tensor imaging several months after injury, even if the patient is no longer symptomatic [18,19]. EMF monitoring may have the potential to provide information regarding immediate or transient alterations in brain EMF activity resulting from mechanical force, especially in cases such as patients with concussion, where there are no structural findings on imaging. This supports the potential use of EMF monitoring as an adjunct neurodiagnostic tool for brain injury localization in real time for inpatients and outpatients, including nonimaging findings of concussion, complementing conventional diagnostic methods such as CT, magnetic resonance imaging, or magnetoencephalography.

The current study is the first to evaluate the efficacy of real-time EMF measurement for specific brain lesion localization in humans. EMF characteristics and EMF stimulation have previously been investigated in swine translational models for TBI [1-3]. In a model of Yucatan minipigs that underwent controlled cortical impact TBI, analysis of EMF data showed differences in preoperative and poststimulation data, reflecting patterns of post-TBI and poststimulation changes occurring due to signal transduction from neural circuits [2]. Furthermore, hyperacute targeted EMF stimulation 20 minutes after injury to the specific area of injury and at a specific frequency was associated with earlier electrophysiologic recovery to baseline EMF patterns preinjury [2]. The neuroprotective and regenerative effects of EMF stimulation have additionally been established, including decreased occurrence of neural apoptosis and increased levels of neuron-specific enolase, which is involved in neurite regeneration through phosphatidylinositol 3-kinase/protein kinase B and mitogen-activated protein kinase/extracellular signal-regulated kinase pathways [2,20]. In subsequent papers, the authors will describe the parameters for EMF modulation through stimulation at the optimal frequency, voltage, and duration.

Limitations

A significant limitation of this study was the small sample size (n = 10). A greater sample size would help to establish the reliability of the localization technique and enable the detection of prognostic factors (i.e., age, comorbidities, and severity of TBI), which may affect patients’ therapeutic response to EMF stimulation. Increasing the number of sensors would permit further localization to areas of interest. Additional standardized testing for cognitive and behavioral changes, as well as the use of advanced imaging techniques, may enable additional correlation to changes in OSs. Other limitations of this study include a single-center exploration, nonrandomized, and nonblinded experimental design, which limits the generalizability of these findings due to lack of randomization and blinding.

## Conclusions

Alterations in neural circuitry in atraumatic and TBI, including concussion, may be measured and precisely localized via continuous and real-time EMF measurements using a portable lightweight helmet for inpatients and outpatients. This localization technique utilizes opposite polarity in OSs to identify SOI and OSs within a specific frequency range of interest. In this study, abnormal brain EMF activity was successfully localized, correlating with neurological deficits on examination and/or structural lesions on CT head and to subclinical symptoms and subradiographic changes, providing an insight into changes in the neuronal cells, neural circuitry, and neuronal pathways. The ability to detect structural and functional brain lesions via EMF recordings supports the potential use of EMF monitoring as an adjunct neurodiagnostic tool for brain injury localization, including nonimaging findings of concussion, complementing conventional diagnostic methods such as CT, MRI, or magnetoencephalography. Further research is required to confirm the efficacy of EMF modulation through specific targeted stimulation as an adjunct to therapy for rescue, recovery, and rehabilitation following brain injury.
